# Vaginal Microbiome-Based Bacterial Signatures for Predicting the Severity of Cervical Intraepithelial Neoplasia

**DOI:** 10.3390/diagnostics10121013

**Published:** 2020-11-26

**Authors:** Yoon Hee Lee, Gi-Ung Kang, Se Young Jeon, Setu Bazie Tagele, Huy Quang Pham, Min-Sueng Kim, Sajjad Ahmad, Da-Ryung Jung, Yeong-Jun Park, Hyung Soo Han, Jae-Ho Shin, Gun Oh Chong

**Affiliations:** 1 Department of Obstetrics and Gynecology, School of Medicine, Kyungpook National University, Daegu 41404, Korea; mylyh3@naver.com; 2 Department of Obstetrics and Gynecology, Kyungpook National University Chilgok Hospital, Daegu 41404, Korea; tpqkf0927@naver.com; 3 Clinical Omics Research Center, School of Medicine, Kyungpook National University, Daegu 41940, Korea; hshan@knu.ac.kr; 4 Department of Applied Biosciences, Kyungpook National University, Daegu 41566, Korea; gukang@knu.ac.kr (G.-U.K.); setubazie@gmail.com (S.B.T.); huypham@knu.ac.kr (H.Q.P.); chahun4270@knu.ac.kr (M.-S.K.); sajjadahmedbot1310@gmail.com (S.A.); yjpark1091@knu.ac.kr (Y.-J.P.); 5 Department of Biomedical Convergence Science & Technology, Kyungpook National University, Daegu 41566, Korea; amugae1210@knu.ac.kr; 6 Department of Physiology, School of Medicine, Kyungpook National University, Daegu 41405, Korea

**Keywords:** cervical intraepithelial neoplasia, machine learning, vaginal microbiome

## Abstract

Although emerging evidence revealed that the gut microbiome served as a tool and as biomarkers for predicting and detecting specific cancer or illness, it is yet unknown if vaginal microbiome-derived bacterial markers can be used as a predictive model to predict the severity of CIN. In this study, we sequenced V3 region of 16S rRNA gene on vaginal swab samples from 66 participants (24 CIN 1−, 42 CIN 2+ patients) and investigated the taxonomic composition. The vaginal microbial diversity was not significantly different between the CIN 1− and CIN 2+ groups. However, we observed *Lactobacillus amylovorus* dominant type (16.7%), which does not belong to conventional community state type (CST). Moreover, a minimal set of 33 bacterial species was identified to maximally differentiate CIN 2+ from CIN 1− in a random forest model, which can distinguish CIN 2+ from CIN 1− (area under the curve (AUC) = 0.952). Among the 33 bacterial species, *Lactobacillus iners* was selected as the most impactful predictor in our model. This finding suggests that the random forest model is able to predict the severity of CIN and vaginal microbiome may play a role as biomarker.

## 1. Introduction

Cervical cancer is common in women worldwide [1], and 95–100% of patients with invasive cervical cancer are infected with human papillomavirus (HPV) [2]. The recognition that HPV is the causative agent of cervical cancer has changed the perception of cervical cancer screening. Compared to primary screening with Pap cytology, the primary HPV test has superior sensitivity for detecting cervical cancer [3]. Although HPV infection is necessarily a cause, it is not determinant of the development of cervical cancer. Most HPV infections are eliminated and only a small fraction of infected women progress to cervical intraepithelial neoplasia (CIN) or cervical cancer [4]. Thus, a persistent infection may not be enough to drive full tumorigenesis. The major risk factors for cervical cancer have been reported to be immune status (immunocompromised), high parity, smoking, combined oral contraceptive use, and other sexually transmitted infections, particularly chlamydia [5,6,7]. More recently, there has been evidence that the vaginal microbiome can either protect or stimulate CIN or cervical cancer progression [8].

Previous studies have demonstrated that the composition of the vaginal microbiome was associated with the acquisition, clearance, or persistence of HPV infection and the severity of CIN [9,10]. With its proven biomarker potential, high diversity microbiome has been frequently shown to have correlation with HPV status and different severity levels of CIN, suggesting that it is a potential predictor for the progression of disease related to HPV infection. Indeed, the presence of *Atopobium vaginae*, *Gardnerella vaginalis*, and *Lactobacillus iners* together with low levels *of Lactobacillus crispatus* was considered as the most hazardous combination for the development of CIN [11]. Moreover, a recent study showed that *Bacteroides fragilis*, *Lactobacillus delbrueckii*, and *Streptococcus agalactiae* have an indirect association with CIN status [12].

A previous study applied a machine learning technique to classify microbial communities according to their bacterial vaginosis characteristics [13]. Furthermore, several attempts have been made to predict diseases, such as nonalcoholic fatty liver, hypertension, and liver cirrhosis, using gut microbiome-based machine learning [14,15,16]. However, machine learning for predicting the severity of CIN based on microbiomes has not yet been reported. Therefore, in this study, we investigated the vaginal microbiome of a well-characterized cohort of participants with biopsy-proven patients in this study. We aimed to develop a panel of vaginal microbiome-derived biomarkers of the severity of CIN using a machine learning-based random forest classification model.

## 2. Materials and Methods

### 2.1. Study Population and Sample Collection

The ethical approval for the present study was acquired by the Institutional Review Board of Kyungpook National University Chilgok Hospital (KNUMC 2015-10-033) (16-11-2015), Republic of Korea. All participants gave written informed consent and experiments performed according to the Declaration of Helsinki. Vaginal smears were collected using pap brush-lines (Bion, Korea) from healthy women and patients with CIN after obtaining approval from our hospital’s Institutional Review Board, and written informed consent from the subjects. HC (Healthy Control) to CIN 1 was defined as CIN 1− and CIN 2+ to cervical cancer was defined as CIN 2+. The brush was placed into the posterior vaginal fornix and rotated 360 degrees clockwise. The samples were transported in DNase, RNase, and pyrogenic-free tubes and immediately stored at −80 °C.

### 2.2. HPV-Assay and HPV Genotyping

HPV genotyping was performed with cervico-vaginal swab specimens using the Anyplex II HPV 28 assay kit (Seegene, Korea). The Anyplex II HPV 28 assay was carried out following the manufacturer’s instructions. Briefly, the five μL of DNA was used in each of 2 20-μL reactions with primer set A or B. In the assay, HPV-specific dual priming oligonucleotides were used for multiplex (real-time) PCR. A total of 28 HPV types were tested to simultaneously detect 18 high-risk types (HPV 16, 18, 26, 31, 33, 35, 39, 45, 51, 52, 56, 58, 59, 66, 68, 69, 73, and 82) and eight low-risk types (HPV 6, 11, 40, 42, 44, 53, 54, and 70).

### 2.3. DNA Extraction and Ion Torrent Sequencing

Bacterial genomic DNA was extracted using the QIAamp PowerFecal Pro DNA Kit (QIAGEN, Germany) according to the manufacturer’s instructions. Bacterial DNA concentration was evaluated using a Qubit^®^ 3.0 Fluorometer (Invitrogen, Waltham, MA, USA) and the isolated DNA was immediately stored at −70 °C until further processing. For the 16S rRNA gene sequencing, the DNA in each sample was amplified with the primers targeting the V3 hypervariable regions of the bacterial 16S ribosomal RNA gene and PCR was performed as described in a previous study [17]. The amplicons were purified using a QIAquick gel extraction kit (QIAGEN, Germany). The purified libraries were pooled in equimolar concentrations and sequenced using the Ion Torrent PGM for 1250 flows with the Ion PGM™ Hi Q Sequencing Kit (Thermo Fisher, Waltham, MA, USA) according to the manufacturer’s instructions.

### 2.4. Bioinformatics Analysis

The amplicon sequencing reads were assessed by FastQC and Trimmomatic [18] used for the pre-processing to acquire the clean reads from raw FASTQ files. We then removed Chimeric sequences, picked operational taxonomic unit (OTU), and performed taxonomic assignment using Quantitative Insights Into Microbial Ecology (QIIME) v1.9.1 [19] and Microbiome Helper package [20]. In order to remove chimeric sequences in our sequences, the script chimera_filter.pl was used based on VSEARCH v1.11.1 [21]. For the OTU level identification, we aligned the processed sequencing reads into OTUs with 97% identity cut-off value with closed-reference and Greengenes database version 13.8. All samples inside of the final OTU table were normalized to equal depths (7231 reads) for the further analysis. To increase bacterial resolution at the species, we further built a custom bacterial identification database that is suitable for the V3 region 16S rRNA gene. Our database is based on the vaginal-microbiota specific database constructed by Fettweis et al. [22], and This database was published in 2012, originally constructed for the taxonomic assignment of hypervariable V1–V3 regions of 16S rRNA gene to show accurate classification at the species level. Several conflicts regarding taxonomic identification between our database and greengenes annotations happened; we then carried out BLAST searches for every OTU with hits at the NCBI nucleotide database and curated manually our database, excluding uncultured microorganisms. Lastly, all OTUs assigned to more than one species followed rules described by Lennard et al. [23].

### 2.5. Data and Statistical Analysis

Differences between subsets were evaluated using Student’s *t*-test, and the differences between proportions were compared using the Chi-square test or Fisher’s exact test. Data analysis was performed on a rarefied OTU table, and the relative and actual abundance were used for all statistical analyses. General analyses of the datasets including principle component analysis (PCA) and non-metric multi-dimensional scaling (NMDS) were conducted in R [24] with tidyr and ggplot2 packages. Phylogenies were manipulated as described by Youngblut et al. [25] and an ANOVA test was applied to test the statistical significance of alpha diversity estimates among groups at different CSTs (Shannon Index and Observed OTUs). The Wilcoxon test was applied for measuring differences in bacterial abundance. A Random Forest (RF) model was constructed to model the severity of CIN based on microbial signatures using the randomForest package in R [26,27]. The abundance of all the bacterial species identified in the CIN 1−and CIN 2+ samples were used as features for building this classifier. The sample sets (24 CIN 1− samples, and 42 CIN 2+ samples) were randomly split into 2 sets (training and test sets). Optimal parameters of the random forest classifier model were obtained through grid search on a five-run 10-fold cross validation procedure using a caret package. The first model was constructed using all the bacterial species variables. Initially, the top 40 discriminating features were obtained based on their importance according to the mean decrease Gini score (indicators of purity by classification splits of given variables). The obtained microbial features were gradually added into the model in order to create the final model. The area under the curve (AUC) of the receiver operating characteristics (ROC) curve was computed to select the best model and it was measured on the test set. To acquire the best threshold in our prediction model, we further analyzed cut-off value, specificity, and sensitivity with bootstrapping method (2000 times).

## 3. Results

### 3.1. Participants’ Characteristics

Sixty-six women in total were included in this study. CIN 1− accounted for 24 women and CIN 2+ for 42 women. The basic characteristics of the study subjects are presented in Table 1. Mean age of the participants in the CIN 2+ group was significantly lower than in the CIN 1− group (42.7 ± 13.2 years vs. 49.2 ± 7.3 years, *p* = 0.0313). However, menopausal status was similar between the two groups (37.5% vs. 26.2%, *p* = 0.3399). HPV infections were detected in seven patients (29.2%) of the CIN 1− group and in 41 patients (97.6%) of the CIN 2+ group (*p* < 0.0001). Moreover, HPV 16/18 was more frequent in the CIN 2+ group than in the CIN 1− group (8.3% vs. 52.4%, *p* = 0.0004).

### 3.2. Vaginal Microbiome

**Figure 1:**
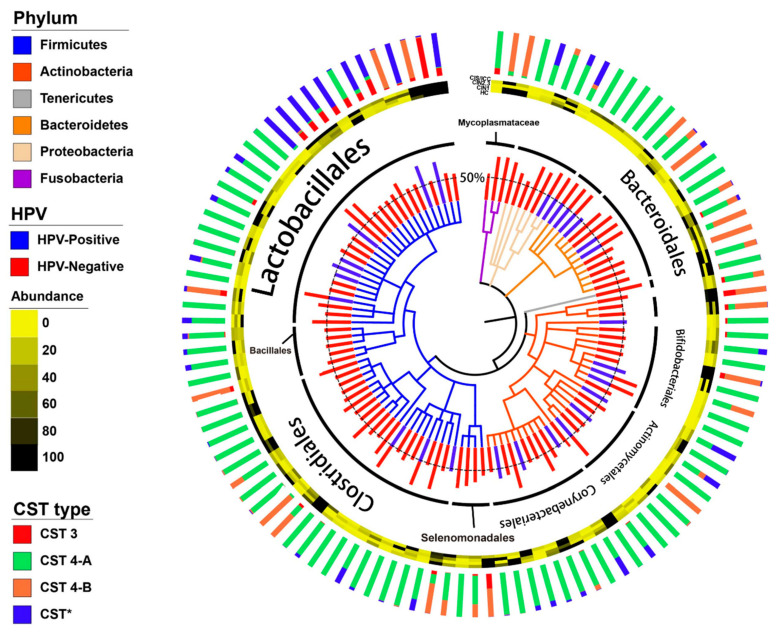
Phylogenetic trees of the vaginal microbiome network by metadata. From outside to inside, the stacked bar chart represents normalized relative abundances of community state type (CST)-related bacterial species; the heatmap represents each clinical stage; the bar charts were enriched in each HPV status, and the trees were grouped according to the phylum level.

**Figure 2:**
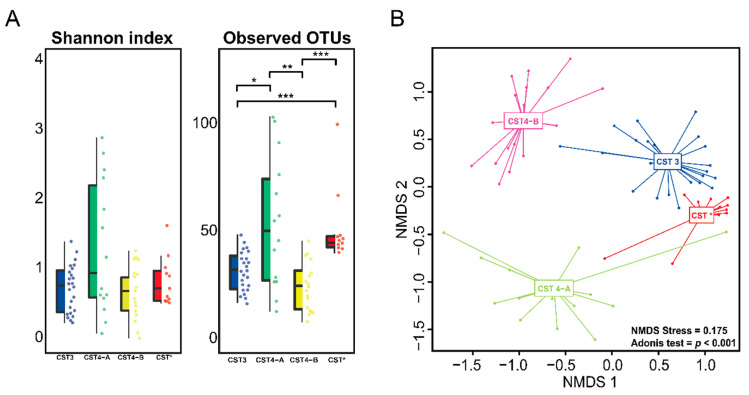
Vaginal microbiome diversity in each CST. (**A**) Alpha diversity including Shannon index and observed operational taxonomic units (OTUs) at the species level. (**B**) Non-metric multidimensional scaling (NMDS) ordination plots based on Bray–Curtis. The microbial communities of each CST type were significantly (Adonis, *p* < 0.001) different from each other. *, *p* < 0.05; **, *p* < 0.01; ***, *p* < 0.001.

Using bacterial amplicon sequencing data, our samples were classified into different bacterial community state types (CSTs) according to the previous description of the vaginal microbiome [28] (Figure 1). The most common CST type was CST 3 (*L. iners*, 36.4%), followed by CST 4 (Diverse group, 47%) (Table 2). Based on the previous classification, CST 4 is grouped into CST 4-A and CST 4-B types. CST 4-A is generally characterized by genera such as *Anaerococcus*, *Corynebacterium*, *Finegoldia*, and *Streptococcus*. CST 4-B, on the other hand, is dominated by *Gardnerella*, *Parvimonas*, *Prevotella*, and *Sneathia* with low proportions of *Lactobacillus* [29]. In our study, out of all participants, samples from eleven participants (16.7%) were dominated by *Lactobacillus amylovorus*, which we proposed as CST* type. Notably, mapping phylum, order, and genus-level relative abundance onto the HPV infection did not demonstrate any clustering of microbiome composition. Vaginal microbiome composition according to the CSTs was evaluated in every sample (CST 3, CST 4-A, CST 4-B, and CST*). Vaginal communities dominated by aerobic bacteria (CST 4-A) had the highest Shannon index and observed OTUs, while in samples where anaerobic bacteria was dominant (CST 4-B), it showed the lowest Shannon index and observed species (Figure 2A). CST classification based on the bacterial species of this cohort showed that the observed clustering trend was significant (NMDS stress = 0.175, *p* < 0.001) (Figure 2B).

### 3.3. Differences between the Vaginal Microbiomes of CIN 1− and CIN 2+

**Figure 3:**
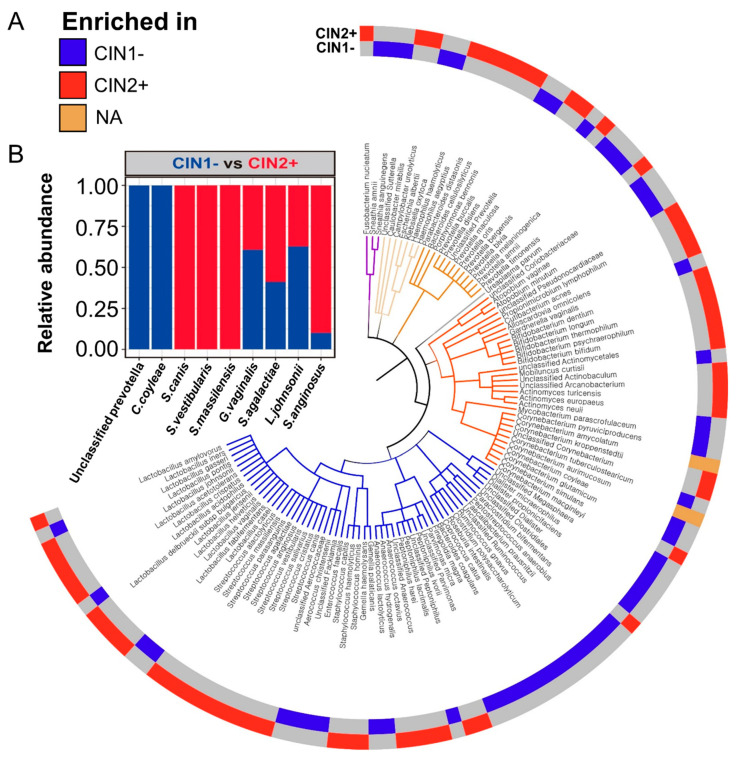
Bacterial differences between CIN stages. The circular phylogenetic tree at the species level is the same as shown in Figure 1. (**A**) The outer circle represents the enriched bacterial species in each CIN and (**B**) the bar charts show mean relative abundance of the most significant species in each CIN stage.

Alpha diversity indices (Shannon index and observed OTUs), which account for the biodiversity of vaginal microbiomes, did not significantly differ according to CIN severity. Principle component analysis (PCA) showed that the full microbial community structure of CIN 1− was not significantly different (*p* = 0.443) from that of CIN 2+ (Figure S1). These results suggest that the full microbial community structures of CIN 1− and CIN 2+ were not distinguishable. Non culture-based analyses of the vaginal microbiome showed different taxonomic compositions according to severity of CIN (Figure 3A). There was no statistical difference between CIN 1- and CIN 2+ at the phylum level and. However, at the species level, Unclassified Prevotella, *Corynebacterium coyleae*, and *Streptococcus canis* were the most significantly different (*p* < 0.05) organisms between CIN 1− and CIN 2+. Most importantly, the relative abundance of the five *Streptococcus* species viz., *S. agalactiae*, *S. anginosus*, *S. canis*, *S. vestibularis*, and *S. massilensis*, and *L. iners*, was higher in CIN 2+ than in CIN 1−. *Lactobacillus johnsonii* was higher in the CIN 1− group compared to the CIN 2+ group (Figure 3B).

### 3.4. Vaginal Microbiome-Derived Signature Can Predict the Severity of CIN

**Figure 4:**
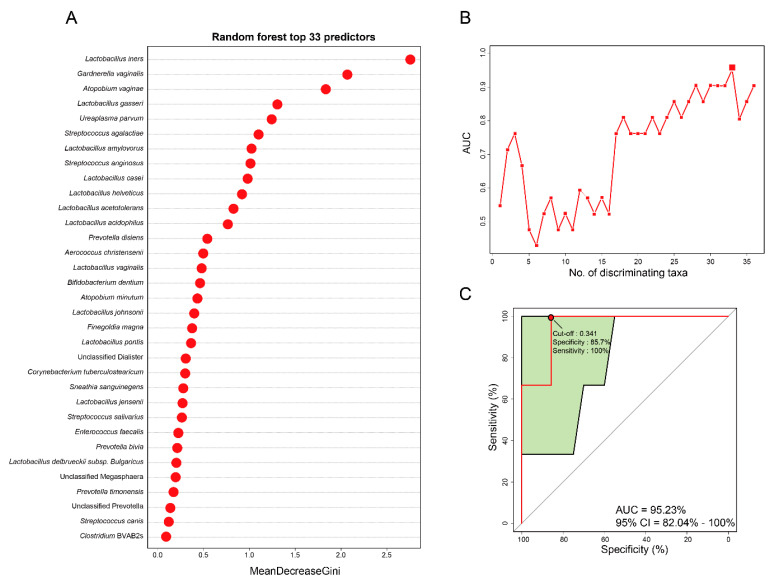
Random forest classifier model using vaginal microbiome-derived bacterial signatures to predict the severity of CIN. (**A**) The 33 most important bacterial species based on their mean decrease Gini scores of the optimal random forest model. (**B**) The 33 bacterial species selected by optimizing the area under the curve (AUC) of the receiver operating characteristics (ROC) curve. (**C**) The AUC of vaginal microbiome-based classification. Random forest classifiers were used to predict CIN 1− and CIN 2+ based on species-level vaginal microbiome composition. The green area of the curve represents the 95% confidence interval (CI) shape.

A Random forest (RF) model was constructed to determine which bacterial species can be potential biomarkers for predicting the severity of CIN. Receiver operating characteristics (ROC) analysis was applied to confirm maximum area under curve (AUC) in order to select specific species as biomarkers and thereby build an optimal model. The highest accuracy was found when 33 bacterial species were selected as optimal marker sets in the random forest model with the lowest number of species (Figure 4A). The optimal 33 features from vaginal samples were selected according to a random forest classifier model to determine whether the model can predict severity of CIN (Figure 4B). The top 33 mean decrease Gini scores of the bacterial species are shown in Figure 4A. The diagnostic value of this model based on those 33 discriminating species was measured according to the AUC and our final model achieved an AUC value of 0.952 (95% CI 0.82–1.00, sensitivity = 1.00, specificity = 0.857, Figure 4C). Among the 33 most impactful bacterial species, *L. iners* was selected as the most discriminating predictor in our model. Also, *L. Johnsonii*, *S. agalactiae*, *S. anginosus*, and *S. canis* were selected as impactful predictors. Overall, these data imply that the selected 33 microbial features suggest further evidence for utilizing vaginal microbiomes as biomarkers and highlight their potential usefulness for detecting the severity of CIN.

## 4. Discussion

Machine learning techniques has been widely used to assess the relationship between microbiome and disease status [30]. However, the potential role and diagnostic accuracy of vaginal microbiome-derived bacterial signatures for predicting CIN severity remains rare. Thus, in this study, we explored several vaginal microbiome-derived bacterial signatures and develop a prediction model using a random forest for predicting the severity of CIN. Vaginal microbiome has previously been classified into six CST levels according to the hierarchical clustering of bacterial species [31], Although our results did not show CST 1, 2, or 5 CST levels. Demographic characterization of each cohort and technical sequencing approaches for identifying bacterial species might have caused this difference [32]. Primers targeting the V4 region of the 16S rRNA gene were used previously [33]. In the present study, on the other hand, we used primer pairs targeting the V3 region, which might contribute to the low abundance of bacterial species such as *L. crispatus*, *L. gasseri*, and *L. jensenii*. Notably, our results showed that *L. amylovorus* was found to be one of the dominant types instead. Similar to the present study, MacIntyre et al. [34] reported that *L. amylovorus* was detected exclusively in Asian women compared to white and black women. Therefore, our finding suggest that since Asian women are more likely to have *L. amylovorus*, it is worth considering *L. amylovorus* as belonging to a new CST classification, CST*.

Most HPV infections are a spontaneous regression due to local immune response. Additional factors might influence the progression of CIN to cervical cancer or its regression. The cervical microbial environment is composed of immune-related cells and its specific microbiota may contribute to the progression of CIN. In the previous studies, prediction of CIN progression was mostly based on HPV infection [35]. However, in this study, we classified the severity of CIN based on bacterial species and we developed a RF model to predict the severity of CIN. Therefore, the preliminary results of this study demonstrated that RF-based ROC analysis can predict the severity of CIN. In addition to this, we described the diagnostic test accuracy of vaginal microbiome-based biomarkers to predict the severity of CIN. Most of the 33 bacterial species used for building each model in this study were directly or indirectly linked to vaginal health [36,37,38,39,40]. Our study showed that the RF model had 33 relevant microbial signatures with differential abundance between CIN 1− and CIN 2+. Furthermore, this model had considerably high accuracy for predicting the severity of CIN (AUC = 0.952) (Figure 4C).

According to our model, *L. iners* was the most impactful predictor in the model (Figure 4). Interestingly, a previous study reported that *L. iners* was a predominant part of the microbial community under the presence of cervical cancer and precancerous lesions in women [41]. Furthermore, the cervical mucosal community dominated by *L. iners* was associated with a higher grade of CIN in women infected with HPV [10]. Additionally, *S. agalactiae* and *S. anginosus* showed high abundance, although non-significant, in CIN 2+ compared to CIN 1−. *S. agalactiae*, also known as Group B *Streptococcus*, is a facultative Gram-positive organism and an important pathogen in aerobic vaginitis. It rarely causes infections in healthy adults; however, it may occasionally cause morbidity in older women, pregnant women, or in patients with underlying predisposing conditions [42]. There are limited studies on the association between *S. agalactiae* and CIN. Only one study showed that *S. agalactiae* was associated with HPV infection and CIN 2+ lesion [12]. Moreover, we observed the highest abundance of *S. anginosus* in the genus *Streptococcus* among patients with CIN2+. Recently, Tao et al. [43] revealed that *S. anginosus* caused the lysis of vaginal epithelial cells. Furthermore, several studies showed the association of certain diseases with *S. anginosus* bacteremia [44,45]. Furthermore, Sasaki et al. [46,47] also reported that the presence of *S. anginosus* was associated with cancers of the upper digestive tracts Thus, our findings contribute important information about the relationships between *L. iners*, *S. agalactiae*, and *S. anginosus*, and the severity of CIN.

Progression of CIN can be detected and predicted with HPV genotyping, proteomics, DNA methylation, or according to the microbial community. The former 3 have been actively studied [35,48,49], but the latter has not been reported yet. HPV testing is used for primary screening of cervical cancer. However, since most HPV infections are harmless, additional triage testing is necessary to properly identify HPV-positive women with a cervical precancerous or cancerous lesion. CIN 2+ has a high probability (30%) of becoming an invasive cancer and detection of CIN 2+ in primary screening is crucial for preventing cervical cancer. Our model showed high accuracy in predicting the severity of CIN. The model would provide a huge benefit for the early diagnosis of women with CIN 2+ and it could be used as a powerful decision-making tool. In this context, it is very plausible that the biomarkers identified in this study could potentially predict the severity of CIN. Predicting the severity of CIN based on the identified biomarkers confers new insights for the prevention of cervical cancer. Hence, we suggest further investigation to quantitatively and qualitatively characterize the identified individual bacterial species and the bacterial community in future studies. The RF method is a machine learning algorithm with the capacity to identify an optimal set of variables with high discriminative power from many dependent or independent variables. The independent signatures can be viewed as a minimized representation of the microbial community for manipulating the dysbiotic microbiome. A few studies used RF machine learning to predict the status of various diseases. Beck et al. demonstrated that machine learning techniques are able to classify microbial communities according to their bacterial vaginosis characteristics with 90 to 95% accuracy [50]. Recent studies also have used the vaginal microbiome as a tool to detect the stage of endometriosis with RF-based machine-learning classification analysis [51]. To the best of our knowledge, the current study is the first study that reports a vaginal microbiome-based RF model for predicting the severity of CIN. Our study built a RF model consisting of 33 relevant bacterial species with differential abundance between CIN 1− and CIN 2+.

In summary, key strength of this current study includes a prospective prediction model, which successfully predicted women at risk of CIN 2+ with a high AUC score. Moreover, we identified 33 bacterial species that are differently abundant and present in both CIN 1− and CIN 2+ from a well-characterized cohort based on the vaginal microbiome. However, we further acknowledge the weakness of our study: (1) The sample size was small and this cohort does not represent the entire Korean population, which have under- or overestimated our prediction model and our study was based on 16S rRNA gene sequencing. Hence, further studies are warranted to increase sample size to validate our prediction model and perform whole metagenome sequencing to fine-tune the prediction model. Therefore, the generalization of this model should be cautious. (2) Our findings only provide preliminary potential of an association between vaginal microbiota and severity of CIN. Therefore, future clinical studies are needed to assess how these bacterial species play a role in vaginal health, affecting the severity of CIN in terms of causality. (3) Future studies should consider performing immunological assay to unravel how hosts interact with its microbiota. (4) We did not evaluate the association between ages and health status. The age difference may be a confounding factor in terms of disease progression. Further studies are essential to evaluate how ages influence the severity of CIN.

## 5. Conclusions

In conclusion, our study suggests the potential associations between vaginal bacterial species and CIN development. Furthermore, utilizing random forest classifiers to predict the severity of CIN can aid patients in the prevention of cervical cancer. This finding may provide an initial step toward exploring the vaginal microbiome for decision-making purposes during CIN development.

## Figures and Tables

**Table 1:** Participants’ characteristics.

Variables	Total (*n* = 66)	CIN 1− (*n* = 24)	CIN 2+ (*n* = 42)	*p* Value
Age (years)	45.1 ± 11.7	49.2 ± 7.3	42.7 ± 13.2	0.0313
Menopause (*n*, %)	20 (30.3)	9 (37.5)	11 (26.2)	0.3399
Marriage (*n*, %)	58 (87.9)	22 (91.7)	36 (85.7)	0.4794
Parity (*n*)	1.7 ± 1.0	1.8 ± 0.9	1.6 ± 1.1	0.4169
Smoker (*n*, %)	4 (6.1)	1 (4.8)	3 (12.0)	0.6139
Contraceptive use (*n*, %)	16 (24.6)	7 (29.2)	9 (22.0)	0.5178
Human papillomavirus (HPV) positive (*n*, %)	48 (72.7)	7 (29.2)	41 (97.6)	<0.0001
HPV16/18 positive (*n*, %)	24 (36.4)	2 (8.3)	22 (52.4)	0.0004

**Table 2:** CSTs according to the severity of cervical intraepithelial neoplasia (CIN).

CST Type	Total (*n* = 66)	CIN 1− (*n* = 24)	CIN 2+ (*n* = 42)	*p* Value
CST3 (*n*, %)	24 (36.4)	9 (37.5)	15 (35.7)	0.8855
CST 4-A (*n*, %)	14 (21.2)	4 (16.7)	10 (23.8)	0.4980
CST 4-B (*n*, %)	17 (25.8)	8 (33.3)	9 (21.4)	0.2911
CST* (*n*, %)	11 (16.7)	3 (12.5)	8 (19.0)	0.4956

CIN = Cervical intraepithelial neoplasia; CST = Community state type; CST3: Primarily composed of *Lactobacillus iners*; CST 4-A; aerobic bacteria such as *Streptococcus* dominated; CST 4-B: *Gardnerella* dominated; CST*: *Lactobacillus amylovorus* dominant type.

## Supplementary Materials

Supplementary materials can be found at https://www.mdpi.com/2075-4418/10/12/1013/s1, Figure S1: Biplot of Principal component analysis (PCA) of the vaginal microbiome according to CIN severity. The PCA plot showed the variation according to severity of CIN. The red circle represents CIN 1−, and the blue triangle represents CI N2+. The arrow indicates the direction of strength of each clinical feature to the overall correlation.

## Abbreviations

CIN	Cervical intraepithelial neoplasia
RF	Random Forest
ROC	Receiver operating characteristic
AUC	Area under curve
CST	Community state type
HPV	Human papillomavirus
