# Supplementary material for: Vaginal Microbiome-Based Bacterial Signatures for Predicting the Severity of Cervical Intraepithelial Neoplasia
Source: Diagnostics (Basel). 2020 Nov 26;10(12):1013. doi: 10.3390/diagnostics10121013 (PMC7761147; doi:10.3390/diagnostics10121013)
Supplement: Supplementary file 1 [file diagnostics-10-01013-s001.pdf]

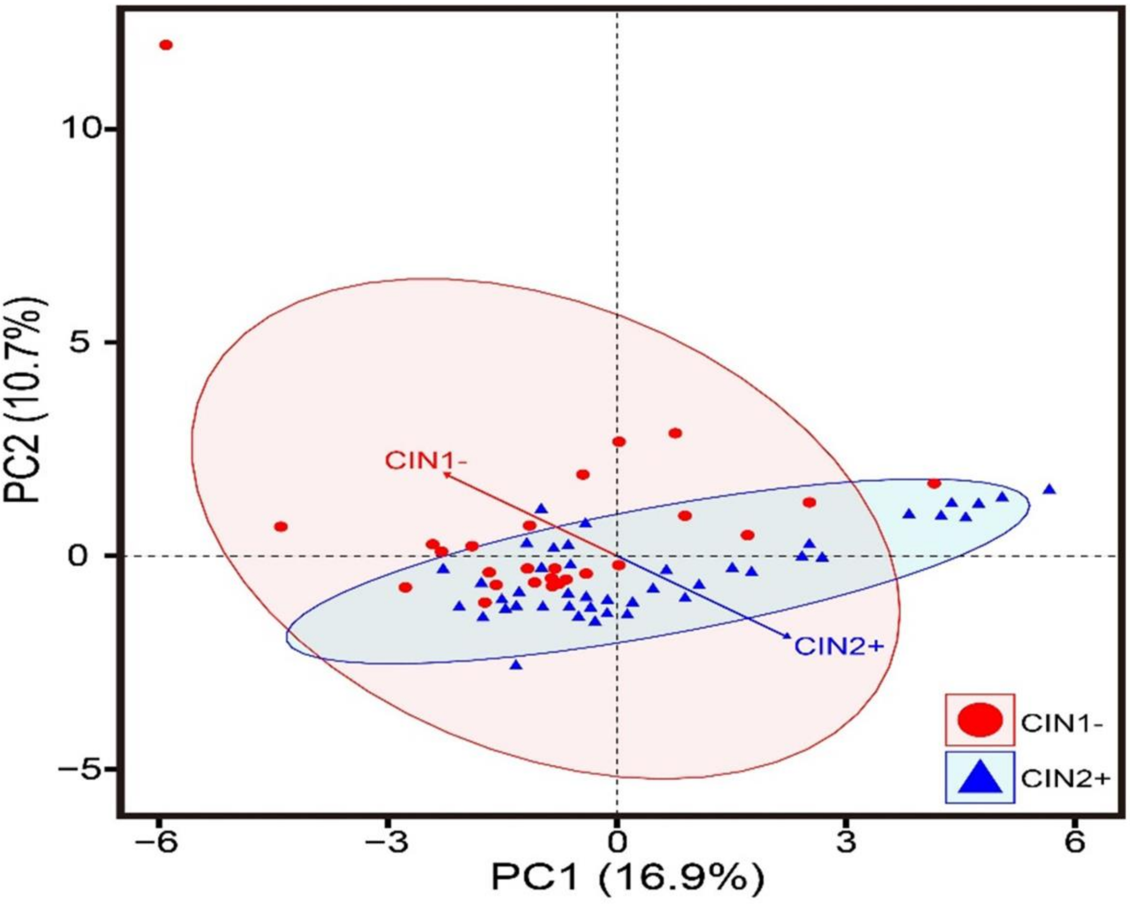

Figure S1. Biplot of Principal component analysis (PCA) of the vaginal microbiome according to CIN severity. The PCA plot showed the variation according to severity of CIN. The red circle represents CIN 1-, and the blue triangle represents CIN 2+. The arrow indicates the direction of strength of each clinical feature to the overall correlation.
